# Risk of Ipsilateral Deep Vein Thrombosis After Kidney Transplantation: A Retrospective Study

**DOI:** 10.7759/cureus.24482

**Published:** 2022-04-25

**Authors:** Abdul Kader Natour, Shahnur Ahmed, Dean Y Kim, Lauren Malinzak, Ali Rteil, Loay Kabbani

**Affiliations:** 1 Vascular Surgery, Henry Ford Health System, Detroit, USA; 2 Transplant Surgery, Henry Ford Health System, Detroit, USA

**Keywords:** risk assessment, laterality, ultrasound (u/s), deep vein thrombosis (dvt), kidney transplantation

## Abstract

Objective: To investigate the incidence and characteristics of deep vein thrombosis (DVT) in kidney transplantation recipients and analyze whether the anatomical side of DVT was associated with the side of the transplanted organ.

Methods: A single-center retrospective medical record review of patients who received a kidney transplant between January 2004 and July 2019 and who subsequently developed DVT. Only patients who received unilateral kidney transplants were included in the study. Patients who underwent concomitant pancreatic transplants, bilateral kidney transplants, or repeat procedures were excluded.

Results: Of the 2449 kidney transplants performed during the study period, 1482 were included in the analysis (948 men [64%]; mean age 61 years). Of 606 duplex ultrasound tests, 115 results confirmed the presence of DVT. The incidence of symptomatic DVT was 4.7%. The most common time of DVT diagnosis was within four weeks after transplantation. Type 2 diabetes, heart failure, acute myocardial infarction, sepsis, chronic obstructive pulmonary disease/abnormal pulmonary function, and being confined to bed were associated with DVT after kidney transplant (all *P* < 0.05). Patients with ultrasound-confirmed DVT had higher mean Caprini scores than patients with negative duplex ultrasounds (*P* < 0.5). Approximately 53% of transplant patients with ultrasound-confirmed DVT had a 1:1 correlation of transplant side to the side of DVT. Cohen kappa statistic 0.03 indicated no correlation between the side of DVT and the side of transplant.

Conclusions: The incidence of DVT after kidney transplant was lower than the incidence reported in the literature. Being confined to a bed may be a risk factor for DVT after transplant surgery. Kidney transplant recipients who had a positive duplex ultrasound had higher Caprini risk assessment scores than transplant recipients who had negative duplex ultrasounds. There was no correlation between the side of the DVT and the side of the transplant.

## Introduction

Deep vein thrombosis (DVT) is a well-recognized complication that occurs in patients as an interplay between vascular endothelial injury, stasis of blood flow, and hypercoagulability [1]. DVT remains an obstacle in patient recovery from various diseases, with a reported case fatality rate of 23% in the first year after diagnosis [2]. Early detection and proper treatment of symptomatic patients significantly reduce mortality [3]. Vascular complications have been reported in 1% to 23% of patients who have had kidney transplantation [4]. In the era of optimizing patient recovery in the postoperative period and reducing hospital-related complications, delineating the incidence, laterality, and predictors of DVT in this group of patients is paramount. A limited number of studies have investigated DVT after kidney transplantation and documented the development of DVT in the perioperative period [5,6]. Caprini et al. [7] identified and categorized risk factors of DVT through the Caprini risk assessment model, which stratifies patients according to the risk of venous thromboembolism (VTE). Whereas the development of vascular complications is well known, little has been reported about the laterality of DVT and the risk factors unique to DVT development after kidney transplantation. This retrospective study aims to investigate the incidence and laterality trends of DVT in patients who have had kidney transplantation and to identify risk factors associated with developing DVT in kidney transplant recipients.

This work has been previously presented at the following meeting: American Venous Forum Annual Meeting, Amelia Island, Florida, March 3-6, 2020.

## Materials and methods

This single-institution retrospective record review study was approved by the Henry Ford Health System Institutional Review Board - Edsel Board (No. 11971) before its initiation, and the need for informed consent was waived.

Patient sample and variables

We retrospectively queried the transplant database at our quaternary care center for all patients who underwent kidney transplants between July 2004 and September 2019. This list was cross-referenced with the vascular surgery database for patients who had duplex ultrasound for DVT during the same time period. Only patients who received unilateral kidney transplants, which was defined as a unilateral abdominal incision made during the operative course, were included in the study. Patients who underwent concomitant pancreatic transplants, bilateral kidney transplants, or repeat procedures were excluded. Patient demographics (age, sex, and race), comorbidities (heart failure, varicose veins, obesity, inflammatory bowel disease, chronic obstructive pulmonary disease, coagulopathies, stroke, sepsis, and diabetes), periprocedural details (location of abdominal incision), and postoperative outcomes (positive duplex ultrasound) were collected from the electronic medical record.

DVT identification

Duplex ultrasonography is considered the gold standard for confirmation of DVT. All DVT imaging was done according to a protocol set by our institution. All symptomatic patients (pain, swelling, skin redness/warmth, or swollen veins) underwent bilateral lower extremity duplex ultrasound. If a common femoral vein (FV) thrombus was visualized or if continuous flow was present, the examination would include the iliac veins. Compression with and without transverse transducer compression in dual screen was done for the lower extremity veins, including the common FV at the level of the junction, proximal FV, profunda femoris vein, mid-FV, distal-FV, mid great sephanous vein, popliteal vein, gastrocnemius vein, posterior tibial vein, and peroneal vein. Spectral waveform demonstrating respiratory variation was done for the common FV, sephanofemoral junction, profunda femoris vein, mid-FV, and popliteal vein. A color Doppler image in the sagittal plane was done if the posterior tibial vein or the peroneal vein were not compressible with transducer compression. Additional images to document areas of suspected thrombus, including the small saphenous vein, soleal veins, and superficial veins, were done when clinically relevant. Upper extremity and central duplex ultrasound results were excluded from the study. Data from the same day of surgery through five years after kidney transplantation were collected from medical records. The time difference between a positive duplex ultrasound to the day of the kidney transplant surgery was used to define the incidence of DVT. The anatomical sides of DVT compared to the side of kidney transplants were used to define laterality. An adaptation of the Caprini risk assessment model was used to define and identify risk factors for DVT development in our patients [7].

Venous thromboembolism prophylaxis

All adult surgical patients received pharmacological prophylaxis for venous thromboembolism (VTE) according to our institutional protocol unless they had a low risk for VTE development or if pharmacological prophylaxis was deemed clinically inappropriate by the provider. It was under the physician’s discretion whether to prescribe VTE prophylaxis (pharmacological or mechanical). Our protocol uses unfractionated heparin in all kidney transplant patients. Intermittent pneumatic compression devices or graduated compression stockings are ordered at the physician’s discretion.

Statistical analysis for DVT laterality

All analyses were performed using SAS 9.4 (SAS Institute Inc., Cary, NC). All continuous variables were reported as mean ± standard deviation, while categorical variables were reported as frequency and column percentages. The main question addressed in this study was whether the lower extremity ipsilateral to the transplanted kidney was at higher risk of developing DVT after transplant surgery. Cohen’s kappa statistic was used to test the level of agreement between the kidney transplant incision and DVT laterality variable and to test our hypothesis that the kidney transplant and DVT sides tended to be the same (the null hypothesis was that there was no agreement [i.e., kappa coefficient = 0]). Statistical significance was set at P < 0.05.

Statistical analysis for DVT risk factors

Each risk factor in the Caprini risk assessment model was defined as a categorical variable. Risk factors were compared in both kidney transplant recipients with either positive or negative duplex ultrasounds. Responses for each risk factor were defined as a continuous variable. For responses in each category, univariate two-group comparisons were performed using independent 2-sample t tests if the variables were normally distributed and Wilcoxon signed-rank tests if the variable was non-normally distributed. For each risk factor, univariate two-group comparisons were performed using chi-square tests when expected cell counts were > 5 and Fisher exact tests when expected cell counts were < 5. Statistical significance was set at P < 0.05.

## Results

Patient characteristics

**Figure 1:**
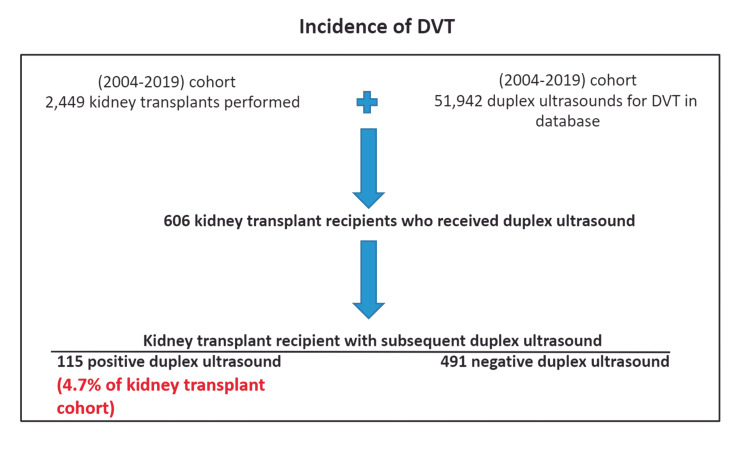
Patient inclusion algorithm.

During the study period, 2449 kidney transplants were performed at our quaternary care center. After exclusion of 967 patients who received either concomitant pancreatic, bilateral, or repeat transplants, 1482 patients met the inclusion criteria (Figure 1). The mean age was 61 years (range 29-86; standard deviation 13 years) (Table 1). Of the 1482 patients, 948 (64%) were men, 800 (54%) were African American, and 534 (36%) were White. Of these 1482 patients, 606 had symptoms indicative of DVT and received bilateral lower extremity duplex ultrasound testing. Of these 606 patients, 115 (19%) had a confirmed DVT (78 unilateral DVT and 37 bilateral DVT). The incidence of DVT in the total cohort was 4.7%.

**Table 1:** Baseline characteristics of the kidney transplant patients who developed deep vein thrombosis SD: standard deviation

Characteristic	Result, N=1482
Age, years, mean ± SD	61 ± 13
Sex, male, N (%)	948 (64%)
Race, N (%)	
African American	800 (54%)
White	534 (36%)
Others	148 (10%)

Analysis of surgical site and DVT laterality

**Figure 2:**
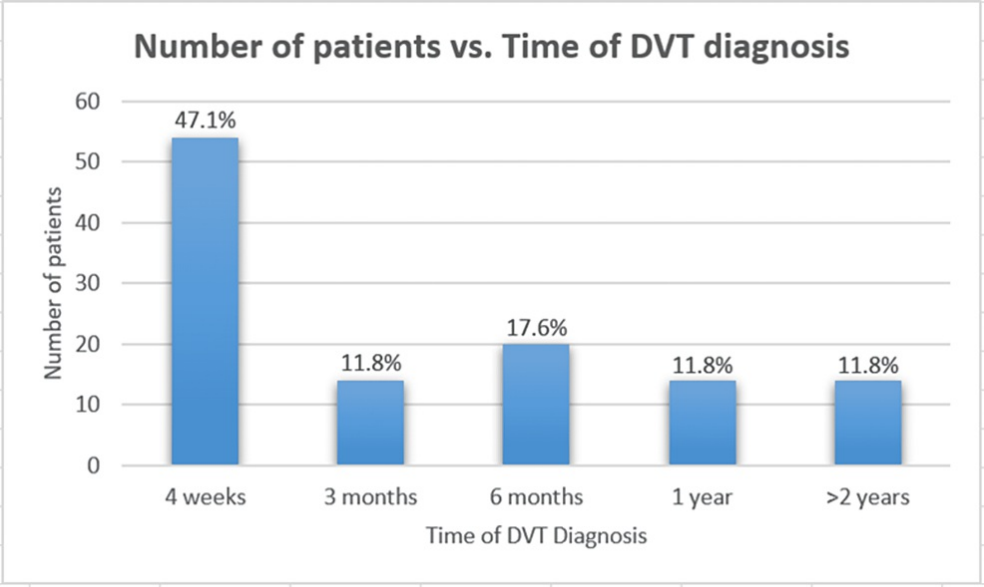
Frequency of kidney transplant patients developing deep vein thrombosis (N=115) relative to time post-transplant. DVT: deep vein thrombosis

Of the patients who had an ultrasound-confirmed unilateral DVT, 41 (53%) had a 1:1 correlation of the transplant side to the side of DVT (Table 2). However, a Cohen kappa statistic of 0.03 (95% CI, -0.09 to 1.15) calculated to assess the correlation of the side of surgical incision to the side of DVT indicated no significant correlation between the side of DVT and the side of the transplant. The most common time of DVT diagnosis was within the first four weeks after transplant surgery (range 0 days to 5 years) (Figure 2).

**Table 2:** Kidney transplant laterality with respect to the laterality of deep vein thrombosis in patients with positive duplex ultrasonography DVT: deep vein thrombosis

Kidney Transplant Surgical Incision	DVT Laterality		Total
	Right	Left	
Right incision	33	2	35
Left incision	35	8	43
Total	68	10	78

Factors associated with risk of DVT

The Caprini risk score was used to assess differences between symptomatic kidney transplant recipients who had positive duplex ultrasound results and patients who were symptomatic but had negative ultrasound results. Type 2 diabetes, heart failure, acute myocardial infarction, sepsis, chronic obstructive pulmonary disease/abnormal pulmonary function, and being confined to bed were significantly associated with having a positive duplex ultrasound test result for DVT (all P < 0.05).

## Discussion

In this study, we showed that the side of kidney transplantation was not associated with the side of DVT development in patients who developed post-transplant DVT. Despite the extensive literature that has addressed the vascular complications associated with kidney transplantation, there is limited information on the laterality of DVT after kidney transplantation, and our study may present a step to fill this knowledge gap. In 1987 a single-center, retrospective study by Allen et al. [5], the authors reported an incidence of DVT of 8.3%. In that study, DVT diagnoses peaked during the fourth month after a kidney transplant. The incidence of DVT after kidney transplantation in our patient population was 4.7%, lower than that reported by Allen et al., and peaked during the first month after kidney transplantation. We calculated incidence per all kidney transplants done during the study period to report the frequency of DVT development regardless of whether patients were symptomatic or not. Our seemingly low incidence may be explained by improved postoperative care that emphasizes early mobility and has better detection with duplex ultrasound with improved ultrasound user dependence. Before 1995, patients with positive D-dimers were considered to have a high clinical likelihood of DVT, and those patients were then assessed with imaging modalities, such as lower extremity venography [8]. Over the last 15 years, new strategies for DVT diagnosis have been introduced, with duplex ultrasonography being the imaging test of choice for DVT diagnosis [9].

Very few studies have documented the laterality of DVT development relative to a medical procedure. Motohashi et al. [10] documented the incidence of DVT as 19.3% in a cohort of 611 patients who had total hip or total knee arthroplasty. Ipsilateral DVT occurrence was 69.5%, which was more common after total knee arthroplasty. Total hip arthroplasty was a separate risk factor in the development of DVT according to the Caprini risk assessment model [7]. In our study, we found that 78 of 115 kidney transplant recipients had unilateral DVT, while 37 had bilateral DVT. Approximately 52.5% of our cohort showed a 1:1 correlation of the side of DVT with the surgical incision side, and patients were more likely to develop right-sided ipsilateral lower extremity DVT; however, this trend was not statistically significant. Thus, the etiology of ipsilateral DVT may be multifactorial, and variables such as surgical site, surrounding anatomical structures, and patient comorbidities may contribute to the site of DVT development.

Other studies also observed that patient age and prolonged bed rest were likely significant risk factors for post-transplant DVT [5,6]. Our study mirrored these findings, showing that being confined to bed, defined as greater than 72 hours, maybe a significant predictor of DVT development. Investigating and delineating the risk factors associated with DVT development after kidney transplantation is critical for learning how best to prevent this dangerous complication, especially during the first month post-transplantation. Thus, risk factors associated with the development of DVT may have significant predictive value in terms of patient morbidity and mortality, and patients with comorbidities such as Type 2 diabetes and cardiovascular disease may require vigilant monitoring for DVT after transplant surgery. The development of DVT is multifactorial and warrants active surveillance with high clinical suspicion in the postoperative setting in the setting of kidney transplant.

Limitations

This article is based on a single-center retrospective study, and conclusions drawn from this study are subject to the limitation of retrospective studies. Causal relationships cannot be determined. Prior studies before 1995 looking at the development of DVT utilized different modalities of a gold standard, including venography, compared to detection in our cohort. However, early mobilization in the surgical intensive care unit may also influence a lower incidence of DVT compared to the literature. Finally, this study was performed at a quaternary care center. Many patients were referred to our center for the sole purpose of undergoing kidney transplantation and did not receive further follow-up.

## Conclusions

The incidence of DVT after kidney transplants in our study was lower than the incidence reported in the literature. Being confined to bed may be a risk factor for DVT after transplant surgery. Kidney transplant recipients who had a positive duplex ultrasound had higher Caprini risk assessment scores than transplant recipients who had negative duplex ultrasounds. Development of DVT was most common in the first month after kidney transplant. The incidence of DVT was not higher on the side of transplantation.
